# SpatialE identifies spatial enrichment of Alzheimer's Disease and Amyotrophic Lateral Sclerosis genes in human brain

**DOI:** 10.1002/alz70855_100010

**Published:** 2025-12-23

**Authors:** Jingyan Guo, Linya You, Yan Chen, Ming Zhang

**Affiliations:** ^1^ Tongji University, Shanghai, Shanghai, China; ^2^ Fudan University, Shanghai, Shanghai, China; ^3^ Clinical Center for Brain and Spinal Cord Research, School of Medicine, Tongji University, Shanghai, 200090, China

## Abstract

**Background:**

Spatial architecture of cell types and gene expression is the foundation of cell‐cell interactions, biological function and disease pathology, but is not well investigated in human cortex, which are highly related to neurodegenerative diseases, such as Alzheimer's disease (AD) and Amyotrophic Lateral Sclerosis (ALS). Recent studies indicated single nucleus transcriptomic features of excitatory neuron vulnerability in AD entorhinal cortex, and motor neuron vulnerability in ALS motor cortex. However, it remains unclear what is the brain regional vulnerability of AD or ALS associated genes.

**Method:**

We developed an entropy‐weighted differential gene expression matrix‐based tool (SpatialE) to identify the spatial enrichment of gene sets in spatial transcriptomics (ST). We benchmarked SpatialE against another enrichment tool (Multimodal Intersection Analysis, MIA) using ST data from human and mouse brain tissues. To investigate regional vulnerability, we analyzed three human motor cortex and two dorsolateral prefrontal cortex (DLPFC) tissues for spatial enrichment analyses of AD and ALS associated genes. We also used Cell2location to estimate the abundance of cell types in AD and ALS related cortex layers.

**Result:**

SpatialE showed more accurate and specific spatial enrichment of regional cell type markers than MIA in both mouse brain and human DLPFC. Spatial transcriptomic analyses of human motor cortex and dorsolateral prefrontal cortex showed heterogenous cell types and spatial gene expression profiles. We found that the expression of AD associated genes (*n* = 139, *n* = 162) that were indicated from two AD genome‐wide association studies are significantly enriched in layer 1 (L1) motor cortex (P_bonferroni<0.01 for 3 ST data), but not in DLPFC. Cell type deconvolution analysis identified abundant expression of astrocytes in human L1 motor cortex. Additionally, the expression of 260 manually curated ALS‐associated genes are significantly enriched in layer 5 (L5) motor cortex and DLPFC (P_bonferroni<0.03 for 5 ST data). Cell type deconvolution analysis revealed an abundant expression of upper motor neurons and L5 excitatory neurons in human L5 motor cortex.

**Conclusion:**

We developed a novel computational tool (SpatialE) to characterize spatial enrichment expression of a gene set in spatial transcriptomics data. Spatial enrichment analyses identified regional brain vulnerability of AD and ALS associated genes in human brain.

